# A Critical Look at the Combined Use of Sulfur and Oxygen Isotopes to Study Microbial Metabolisms in Methane-Rich Environments

**DOI:** 10.3389/fmicb.2018.00519

**Published:** 2018-04-06

**Authors:** Gilad Antler, André Pellerin

**Affiliations:** ^1^Department of Earth Sciences, University of Cambridge, Cambridge, United Kingdom; ^2^Department of Geological and Environmental Sciences, Ben-Gurion University of the Negev, Beersheba, Israel; ^3^The Interuniversity Institute for Marine Sciences, Eilat, Israel; ^4^Center for Geomicrobiology, Department of Bioscience, Aarhus University, Aarhus, Denmark

**Keywords:** sulfur isotopes, oxygen isotopes, anaerobic oxidation of methane (AOM), sulfate reduction rates, marine sediments

## Abstract

Separating the contributions of anaerobic oxidation of methane and organoclastic sulfate reduction in the overall sedimentary sulfur cycle of marine sediments has benefited from advances in isotope biogeochemistry. Particularly, the coupling of sulfur and oxygen isotopes measured in the residual sulfate pool (δ^18^O_SO4_ vs. δ^34^S_SO4_). Yet, some important questions remain. Recent works have observed patterns that are inconsistent with previous interpretations. We differentiate the contributions of oxygen and sulfur isotopes to separating the anaerobic oxidation of methane and organoclastic sulfate reduction into three phases; first evidence from conventional high methane vs. low methane sites suggests a clear relationship between oxygen and sulfur isotopes in porewater and the metabolic process taking place. Second, evidence from pure cultures and organic matter rich sites with low levels of methane suggest the signatures of both processes overlap and cannot be differentiated. Third, we take a critical look at the use of oxygen and sulfur isotopes to differentiate metabolic processes (anaerobic oxidation of methane vs. organoclastic sulfate reduction). We identify that it is essential to develop a better understanding of the oxygen kinetic isotope effect, the degree of isotope exchange with sulfur intermediates as well as establishing their relationships with the cell-specific metabolic rates if we are to develop this proxy into a reliable tool to study the sulfur cycle in marine sediments and the geological record.

## Introduction

Methane is an important greenhouse gas which moderates the climate of the planet and marine sediments are Earth’s largest methane reservoir and production site (Whiticar et al., 1986; Kvenvolden, 1988). In the distant past, fluxes of methane from marine pelagic environments may have triggered abrupt warming periods (Dickens et al., 1995) and understanding the controls on its production and release is of interest for predictions of climate change and reconstruction of Earth’s past. A continuous flux of methane from biotic and thermogenic origins diffuses or escapes from production zones toward the surface. The distinct zone where upward diffusion of methane meets with the downward diffusion of sulfate from seawater is called the sulfate methane transition zone. This zone is where the anaerobic oxidation of methane by sulfate (hereafter ‘AOM-SR’) is catalyzed by consortia of bacteria and archaea who derive energy for growth by reacting the two together (Boetius et al., 2000). This leads to the quasi-quantitative consumption of both substrates, the establishment of steady state concentration profiles and the oxidation of a large fraction of the flux of methane, which would otherwise diffuses upward into the ocean (Wuebbles and Hayhoe, 2002). Sulfate is also consumed by sulfate reducing microorganisms which couple the oxidation of organic matter deposited on the seafloor with the reduction of sulfate (hereafter organoclastic sulfate reduction – OSR).

Separating the contributions of OSR and AOM-SR to the overall sedimentary sulfur cycle has been challenging but has benefited from recent advances in isotope biogeochemistry. Stable carbon isotopes have been vastly used to address these processes. Recently, the coupling of sulfur and oxygen isotopes measured in the residual sulfate pool (δ^18^O_SO4_ vs. δ^34^S_SO4_) has also been demonstrated to be instrumental in our understanding of the interactions between the carbon and sulfur cycles. Metabolic processes discriminate between light and heavy isotopologues and the progressive enrichments in heavy isotoplogues observed in the residual sulfate pool can trace this activity. Oxygen and sulfur isotopes are sensitive to the reductive pathway and geochemical conditions under which multiple biological and abiotic reactions occur (Brunner et al., 2005; Turchyn et al., 2006; Wortmann et al., 2007). Yet, similar to other proxies, the combined S-O isotopes in porewater sulfate is propagating on Elderfield’s proxy confidence curve [from the ‘Optimism phase,’ through the ‘Pessimism phase,’ to the ‘Realism phase’–(Elderfield, 2002)]. With recent works observing δ^18^O_SO4_ vs. δ^34^S_SO4_ patterns that are inconsistent with previously held interpretations, we ask: what are the limits of application of the combined sulfur–oxygen isotope tool to distinguish between AOM-SR and OSR?

## The Optimism Phase

**FIGURE 1:**
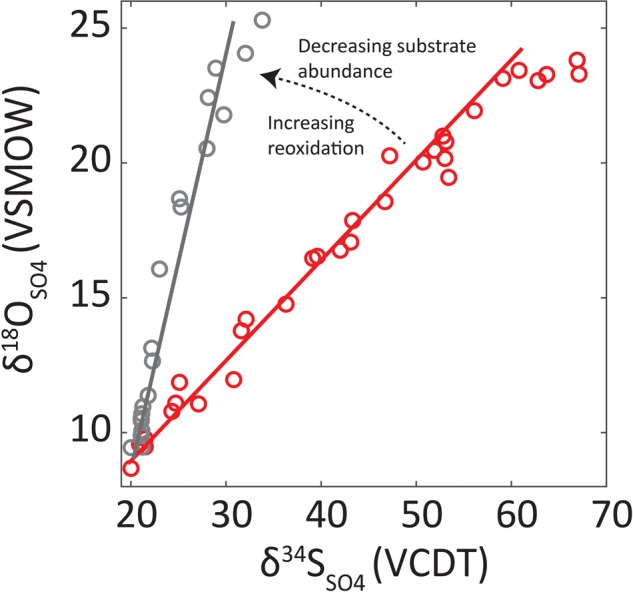
Graphical demonstration of the slope of the apparent linear phase (SALP) and its interpretation. Red line is SALP associated with seep environments while black line is associated with OSR-dominated deep sea sediments. Red circles are data from seeps, black circles from deep sea sediments (Antler et al., 2015). The decreasing substrate refers to the decrease in electron donor supply and ‘Increasing reoxidation’ refers to the increase in reduced sulfur which is oxidized to sulfate.

In a given pore water profile, the slope of the tangent on the δ^18^O_SO4_ vs. δ^34^S_SO4_ (slope of the apparent linear phase—SALP) is related to the overall sulfate reduction rate (SRR) where lower rates lead to steeper SALP (see **Figure 1**) (Böttcher et al., 1998, 1999; Aharon and Fu, 2000; Brunner et al., 2005; Antler et al., 2013). The negative relationship between SALP and SRR is understood as increased reversibility of the sulfate reducing enzymatic pathway (Fritz et al., 1989; Brunner and Bernasconi, 2005). The SALP is also affected by sulfur cycling extracellularly where waste products of sulfate reduction can be reoxidized. For example, disproportionation will lead to higher SALP values (Böttcher and Thamdrup, 2001; Böttcher et al., 2001, 2005; Blonder et al., 2017). These relationships are utilized to study the sulfur cycle in marine sediments.

**FIGURE 2:**
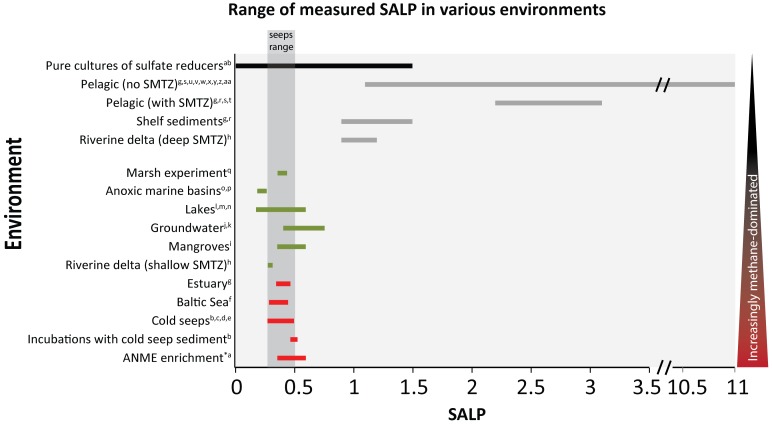
Slope of the apparent linear phase (SALP) measured in various environments. Note the broken axis between 3.5 and 10.5. Black bar indicates pure cultures of dissimilatory sulfate reducers, gray bars specify environments where methane is thought to play a minor role according to the reference. Green bars indicate environments where the presence and contribution of methane to the overall removal of sulfate is questionable. Red bars indicate environments that are thought to be methane dominated. Gray dashed line indicates the SALP measured in methane seeps. Triangle grading from black to red is meant to demonstrate the overall increase in methane in that specific environment. Data (Deusner et al., 2014)^a^, (Sivan et al., 2014)^b^, (Aharon and Fu, 2000)^c^, (Aharon and Fu, 2003)^d^, (Rubin-Blum et al., 2014)^e^, (Strauss et al., 2012)^f^, (Antler et al., 2013)^g^, (Aller et al., 2010)^h^, (Crémière et al., 2017)^i^, (Avrahamov et al., 2014)^j^, (Einsiedl et al., 2015)^k^, (Knossow et al., 2015)^l^, (Gilhooly et al., 2016)^m^, (Antler, 2012)^n^, (Gomes and Johnston, 2017)^o^, (Mandernack and Skei, 2003)^p^, (Mills et al., 2016)^q^, (Turchyn et al., 2006)^r^, (Turchyn et al., 2016)^s^, (Riedinger et al., 2010)^t^, (Böttcher et al., 1998)^u^, (Böttcher et al., 1999)^v^, (Böttcher et al., 2006)^w^, (Blake et al., 2006)^x^, (Turchyn et al., 2006)^y^, (Turchyn et al., 2016)^z^, (Wortmann et al., 2007)^aa^, (Antler et al., 2017)^ab^. ^∗^Indicates SALP values are calculated.

In pelagic regions of the oceans, where the sedimentation rate and supply of organic carbon is relatively low, most of the organic matter remineralization occurs through OSR and only a small fraction of mineralization is via methanogenesis. Under these conditions, sulfate reduction proceeds under organic matter limitation (Glombitza et al., 2015). This results in pore water profiles characterized by high SALP numbers (**Figure 2**, gray bars). On the other hand, sites with a high organic carbon flux to the seafloor results in excess methane diffusing upward and leads to low SALP numbers. In fact, methane-rich environments interestingly fall in a very narrow range of SALPs (see highlighted ‘seeps’ range, **Figure 2**). AOM-SR-dominated sites record maximum SALP values of ∼0.4 (**Figure 2**) and reflect the fact that these environments are not electron donor (methane) limited and thus have low reversibility (Antler et al., 2015). Indeed, low SALP appears to be universal for methane rich environments, regardless of the ambient temperature, water depth (or pressure), salinity and sedimentation rate (Antler et al., 2015; Feng et al., 2016). Therefore, it appears SALP may distinguish between OSR and AOM-SR, even when sulfate concentration profiles are similar in their sulfate penetration depth (Antler et al., 2014). Overall, this tool seems to be robust under different environmental conditions and a great potential for both modern and geological applications.

## The Pessimism Phase

However, observations of low SALP in organic-rich, OSR-dominated sediments of marine mangroves (slope of 0.36 ±0.06; Crémière et al., 2017) questions the robustness of SALP to identify AOM-SR dominated environments. This suggests that low SALPs are not unique to AOM-SR in the environment. (**Figure 2**; green bars). To address this issue, we compiled SALP data from sites that are likely to be AOM-SR dominated as well as sites that are OSR dominated (**Figure 2**, red bars and green bars, respectively). It appears there is no significant difference in SALP between AOM-SR and some OSR dominated environments. This means that the SALP does not provide sufficient information to distinguish between these two processes.

Pure cultures of sulfate reducing microorganisms (**Figure 2**, black bar) are analogous in metabolic pathway to OSR in marine sediments and are useful to discuss the limits of SALP associated with this metabolism. They have been demonstrated to produce the entire range of SALPs observed in nature with the exception of pelagic sites (**Figure 2**; gray bars). In pure culture, a near zero SALP indicates the sulfur isotope discrimination of sulfate reduction is being expressed but not the oxygen isotope effect. An absence of oxygen isotope enrichment but presence of sulfur isotope enrichment can be explained if there is no kinetic isotope effect for oxygen, no back reaction of sulfur intermediates (only the entry of sulfate into the cell is reversible) or if the turnover times of the intracellular intermediate pools where oxygen equilibration can happen are so short that it simply is not expressed. Sulfite is the main sulfur intermediate which enables oxygen isotope exchange and does so in a matter of minutes (Betts and Voss, 1970; Horner and Connick, 2003; Müller et al., 2013; Wankel et al., 2014). Recent theoretical work on intracellular APS and sulfite concentrations has predicted sub-micromolar and millimolar levels respectively (Wing and Halevy, 2014) while measurements in pure cultures grown in batch suggest concentrations of around a hundred micromolar and ten micromolar respectively (Sim et al., 2017). In the first instance, (Wing and Halevy, 2014) assumed the reduction of APS and sulfite is coupled to the oxidation of menaquinone (a mildly reducing electron donor), forcing a high ratio of reduced to oxidized menaquinone to generate a favorable redox potential. This produces a low APS to sulfite ratio of 1:1000 and relatively high sulfite concentrations. Ultimately this results in the intracellular sulfite pool having a large turnover time and enable high oxygen isotope exchange. Contrarily, the measurements of (Sim et al., 2017) in (fast growing) batch cultures suggest the ratio of APS to sulfite is ∼10:1 with both having relatively low concentrations resulting in a fast turnover time and therefore, less oxygen isotope exchange. A calculation of the turnover times of sulfur within a cell under conditions which produce SALP close to 0 [cell-specific sulfate reduction rate ≈50 fmol/cell/day and assumptions on cellular and intracellular parameters; cell size = 1.5 × 10^-18^ m^3^, internal adenosine 5′ phosphosulfate concentration = 0.1–0.001 mM, internal sulfite concentration = 10 μM–10 mM (Wing and Halevy, 2014; Sim et al., 2017)] suggests the APS pool is turned over within a fraction of a second (<<1 s) and the sulfite pool in 0.03–30 s. In these conditions only partial oxygen isotope exchange can occur between sulfite and water. Naturally, as the cell specific sulfate reduction rates in the environment are 2–6 orders of magnitude lower than this calculation, e.g., (Holmkvist et al., 2011), turnover of intracellular metabolites increases from seconds to minutes or hours and the oxygen isotope effect becomes significant. These findings are also in agreement with the finding that low csSRR which produce large sulfur isotope fractionations (such as the ones observed in the environment) can only be driven by modestly negative electron carriers (i.e., menaquinone) whereas very negative electron carriers (i.e., ferredoxins) restrict the net fractionation to around < 22‰ (Wenk et al., 2017). In most cases, SALP is a direct consequence of these intracellular states. In addition, it is possible that the abundance of different sulfoxy isomers might have an effect on the rate of the isotopic exchange with water as well as on the magnitude of isotope fractionation (Müller et al., 2013; Wankel et al., 2014). These should be incorporated into a kinetic-thermodynamic model as it would enable oxygen isotope exchange to be quantified at the cellular level and be an important tool to predict metabolite cycling within sulfate reducing organisms. For environmental studies, it would allow testing whether cellular-level processes associated with OSR are a fair approximation of environmental observations. This should be a priority of future work.

## The Realism Phase

Empirical studies on AOM-SR have shown a high dependence of sulfur and oxygen isotope fractionation on methane concentrations (Deusner et al., 2014). Unlike pure cultures of sulfate reducing microorganism, AOM-SR is fundamentally incapable of having a zero contribution from δ^18^O in water. In AOM-SR, consortia of archaea and proteobacteria reduce sulfate to sulfide but they do so by disproportionating intermediate sulfur species (Milucka et al., 2012). This leads to the regeneration of 1/8^th^ of the reduced sulfate, and an ‘inescapable’ generation of sulfate with a partial contribution from the isotopic signature of water. However, the oxygen isotope enrichment gained by disproportionation only account partially for the inability of AOM-SR to produce SALP below 0.25. This shunt in the sulfur cycle in AOM-SR accounts for only about 12% of the total sulfur and is not enough to produce the characteristic range of SALP seen methane-dominated environments (Antler et al., 2015). Contrarily to OSR, AOM-SR requires a degree of reversibility which allows the oxygen isotope exchange of intermediates to be expressed in the sulfate pool even at the highest possible metabolic rates (no methane or sulfate limitation). The fact that the range of SALPs observed in AOM-SR is so narrow is something which remains thus far a perplexing issue that will require further empirical work.

The linear progression of δ^18^O_SO4_ vs. δ^34^S_SO4_ in nature (slope of about 0.25) is mostly interpreted as an indicator for the ratio between the kinetic isotope effects of δ^34^S_SO4_ and δ^18^O_SO4_, where the oxygen fractionation factor is roughly ¼ of that of sulfur (Mizutani and Rafter, 1969). However, there is no solid evidence for an oxygen kinetic isotope effect in pure cultures of sulfate reducing microorganisms. In fact, pure culture experiments with low SALPs similar to some natural environments and smaller (SALP = 0.25) already show dependence on the oxygen isotope composition of water (Antler et al., 2017), suggesting that the kinetic isotope effect for oxygen isotopes is small or even negligible. Porewater SALPs are evidently more complex than simple kinetic isotope fractionation or equilibrium isotope exchange. An alternative way to reconcile the linear sulfur vs. oxygen isotopes profiles in nature with a small kinetic isotope effect for oxygen may be that SALP is generated by diffusion between two end members, such as seawater sulfate and the enriched (δ^34^S_SO4_) and fully equilibrated (δ^18^O_SO4_) pool of sulfate near the sulfate methane transition zone.

In environmental samples, determining if a given SALP is produced by AOM-SR or OSR is challenging for multiple reasons. First, the majority of published work on δ^18^O_SO4_ and δ^34^S_SO4_ does not provide independent, robust evidence of the metabolic processes (AOM-SR or OSR) controlling the pore water profiles. Second, a spatial separation of OSR and AOM-SR at different depths can effectively hide the AOM-SR signature. For example, an abundance of pelagic sites such as from IODP expeditions clearly have a sulfate methane transition zone where sulfate and methane are both consumed. However, OSR in the surface sediment drives δ^18^O_SO4_ to equilibrium before AOM-SR has a chance to imprint its signature on the oxygen isotopes. Third, the level of reoxidation occurring in sediments plays an important role in SALP as it increases recycling of the δ^18^O_H2O_ signature to δ^18^O_SO4_, resulting in a steepening of SALP. Estimating the contribution of reoxidation to the overall sulfur cycle is a large uncertainty in the marine sedimentary sulfur cycle (Jørgensen and Nelson, 2004) and an important aspect of the sulfur cycle where SALP can be a critically important tool, e.g., Brunner et al. (2016).

There is a potential to preserve the SALP signature in sulfate containing minerals, such as barite and celestine and in carbonates (as carbonate associated sulfate) and that it may be a useful indicator of past methane seeps (Feng et al., 2016). Among the three, carbonates are by far more spatially and temporally abundant in the geological record. Authigenic carbonates are more likely to form during AOM-SR due to the reaction stoichiometry, which results in sharp increases in pH (Soetaert et al., 2007). Therefore, although the SALP cannot distinguish between AOM-SR and high activity OSR, high activity OSR is not associated with extensive carbonate precipitation like AOM-SR.

High carbon isotope fractionation during methanogenesis results in the δ^13^C of the methane being as low as -100‰ (Whiticar, 1999). Since AOM-SR consumes this isotopically light carbon while OSR does not, AOM-SR results in the production of δ^13^C -depleted dissolved inorganic carbon, a marker which can be utilized in a quantitative way to estimate the contributions of AOM-SR and OSR to the DIC pool in marine sediments (Martens et al., 1999; Sivan et al., 2007; Malinverno and Pohlman, 2011; Komada et al., 2016). For example, δ^13^C and Δ^14^C of the major carbon pools in the Santa Barbara Basin were used to study the discrepancy of methane and sulfate fluxes to the SMT. Based on the carbon isotope signatures in the resulting DIC pools, OSR could account for 35–45% of the sulfate consumption in the sulfate methane transition zone (Komada et al., 2016). Such quantitative work, while tedious, is an effective way to differentiate AOM and OSR, which could be utilized in conjunction with S–O isotopes to further constrain the nature of carbon mineralization in sediments. Since C–S–O isotopes are preserved over geological timescales (as carbonates and carbonate-associated suflates), they should also be useful when applied to the geological record. Isotopically light carbon isotope in lipids have long been known to be a reliable tracer of methanotrophy because of the distinctly light isotope signature of methane (Hinrichs et al., 1999) and carbon isotopes of carbonates associated with AOM-SR are usually light, a tell tale marker of the methanotrophy associated with AOM-SR (Drake et al., 2015). From a quantitative perspective, these markers have limitations because unknown contributions of carbon from non-methane sources can be incorporated. Non-isotopic quantitative approaches to estimating contributions from AOM-SR and OSR also hold significant promise. For instance, careful comparisons of the fluxes of methane and sulfate into the SMT, combined with in-situ AOM-SR radiotracer incubation experiments e.g., (Beulig et al., 2017) may be able to estimate the relative importance of AOM-SR and OSR in the SMT. A tool which previously held high hopes was molecular approaches to identify microbial community abundances and relate them to process rates, e.g., (Carolan et al., 2015). However, these have thus far been unsuccessful at quantitatively differentiating AOM-SR and OSR. This is in large part because there exists phylogenetic and functional overlap between oxidizing and reducing reactions; for instance ANME and methanogens, e.g., (McGlynn, 2017) or sulfide oxidizers and sulfate reducers, e.g., (Thorup et al., 2017) which makes it impossible to ascertain which organism is doing what in the subsurface. Molecular approaches are unlikely to yield quantitatively useful information by themselves until significant progress is made in connecting genotypes to metabolic functions.

SALP provides a powerful tool to study the sulfur cycle, by sensitively responding to changes in the reduction of sulfate vs. the oxidation of reduce sulfur compounds. In this contribution, we explored the limitation of this tool as a way to distinguish between AOM-SR and OSR. Because of the multiple parameters which control the extent of SALP, the information provided and what it tells of the microbial sulfur transformations are not generally applicable to all circumstances. However, with a careful site-specific approach and in combination with other proxies, SALP can contribute vital information on the microbial and chemical reactions taking place in marine sediments. Further refining (1) our understanding on the magnitude of the oxygen isotope kinetic fractionation during the APS reduction to sulfite, (2) the degree of oxygen isotope exchange between sulfur intermediates and water and (3) its relationship to the cell specific sulfate reduction rate, would greatly improve our ability to interpret the SALP as an indicator of the microbial transformations taking place. Doing so will transform this analytical tool into a reliable proxy of sulfur cycling.

## Author Contributions

Both authors contributed to the redaction of the manuscript and figures.

## Conflict of Interest Statement

The authors declare that the research was conducted in the absence of any commercial or financial relationships that could be construed as a potential conflict of interest.
